# The clinical relevance of OSM in inflammatory diseases: a comprehensive review

**DOI:** 10.3389/fimmu.2023.1239732

**Published:** 2023-09-29

**Authors:** Cody L. Wolf, Clyde Pruett, Darren Lighter, Cheryl L. Jorcyk

**Affiliations:** ^1^ Department of Biomolecular Sciences, Boise State University, Boise, ID, United States; ^2^ Department of Biological Sciences, Boise State University, Boise, ID, United States

**Keywords:** oncostatin M (OSM), oncostatin M receptor beta (OSMRβ), inflammatory diseases, cytokine, cancer, metastasis, drug development, therapeutics

## Abstract

Oncostatin M (OSM) is a pleiotropic cytokine involved in a variety of inflammatory responses such as wound healing, liver regeneration, and bone remodeling. As a member of the interleukin-6 (IL-6) family of cytokines, OSM binds the shared receptor gp130, recruits either OSMRβ or LIFRβ, and activates a variety of signaling pathways including the JAK/STAT, MAPK, JNK, and PI3K/AKT pathways. Since its discovery in 1986, OSM has been identified as a significant contributor to a multitude of inflammatory diseases, including arthritis, inflammatory bowel disease, lung and skin disease, cardiovascular disease, and most recently, COVID-19. Additionally, OSM has also been extensively studied in the context of several cancer types including breast, cervical, ovarian, testicular, colon and gastrointestinal, brain,lung, skin, as well as other cancers. While OSM has been recognized as a significant contributor for each of these diseases, and studies have shown OSM inhibition is effective at treating or reducing symptoms, very few therapeutics have succeeded into clinical trials, and none have yet been approved by the FDA for treatment. In this review, we outline the role OSM plays in a variety of inflammatory diseases, including cancer, and outline the previous and current strategies for developing an inhibitor for OSM signaling.

## 1 Introduction

**Figure 1:**
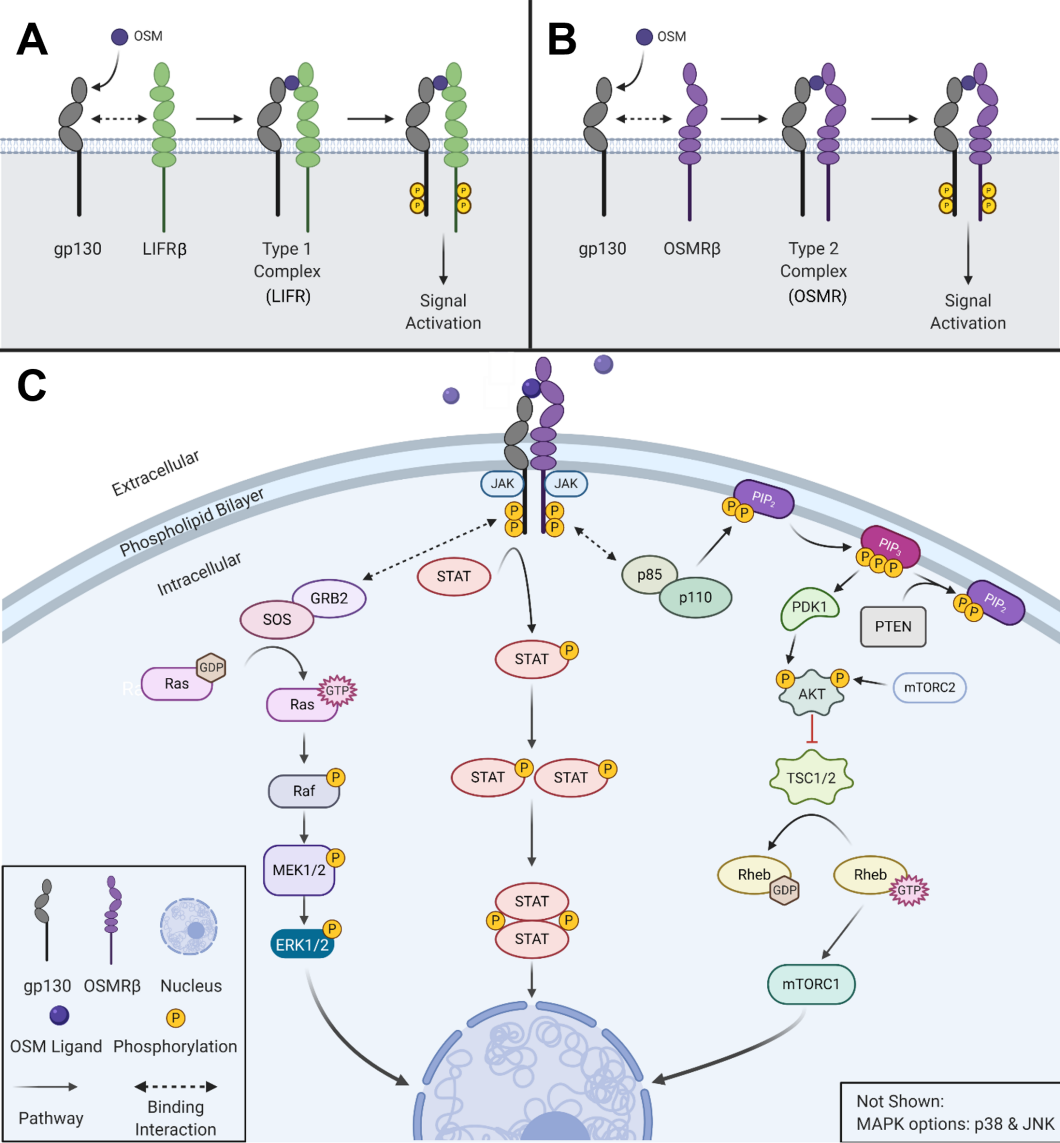
OSM activates multiple signaling cascades. **(A)** OSM binds to gp130 and then recruits LIFRβ to form a type I complex (LIFR). **(B)** OSM binds to gp130 and then recruits its major receptor complex subunit OSMRβ to form a type II complex. **(C)** Visual representation of the signaling pathways used by OSM type II complex. Created with BioRender.com.

Oncostatin-M (OSM) is an interleukin-6 (IL-6) family cytokine first isolated in 1986 from human histiocytic lymphoma U937 cells (1). It was initially identified as a cytostatic protein for melanoma cells, thus deriving its name (‘onco’ for cancer and ‘statin’ for inhibitor) (1). Other than OSM, the IL-6 family members consist of the parent protein IL-6, leukemia inhibitory factor (LIF), IL-11, IL-27, cardiotrophin-1 (CT-1), ciliary neurotrophic factor (CNTF), and cardiotrophin-like cytokine factor 1 (CLCF1). The human *OSM* gene encodes for a 2 kb mRNA transcript that is translated and cleaved into a soluble 227 amino acid pro-OSM polypeptide with a 28 kDa molecular weight (2). Mature OSM is synthesized after a C-terminal cleavage of 31 amino acids, yielding a 196 amino acid, ~22kDa protein (3, 4). OSM, like all other IL-6 family members, has a crystal structure consisting of a four alpha-helical up-up-down-down configuration (5). LIF and OSM are structurally and genetically the most similar members of the IL-6 family, resulting from an ancestral gene duplication event (6). Similar to other IL-6 family cytokine members, OSM utilizes the shared receptor protein membrane glycoprotein 130 (gp130; also known as IL-6Rβ) and a unique receptor protein to create a complete signaling receptor complex (7–10). In the case of OSM, it possesses the capability to interact and transduce signaling through two separate complexes (7–10). OSM first binds to the extracellular cytokine-binding homology region (CHR) domain of gp130 with a high affinity (~10^-8^ M) and subsequently recruits either the leukemia inhibitory factor receptor beta (LIFRβ) or oncostatin M receptor beta (OSMRβ); to form either a type I or type II receptor complex (LIFRβ/gp130 and OSMRβ/gp130, respectively) (**Figures 1A, B**) (11–16). While the OSMR (type II) complex has been studied extensively in various human cell lines, it remains unclear how OSM interacting with the LIFR (type I) complex affects signaling or disease progression; however, recent research has indicated that OSM binds to LIFR with significantly lower affinity than its specific receptor (17, 18). After binding to the OSMR complex, several signaling pathways are activated, including the Janus-activated kinase/signal transducer and activator of transcription 3 (JAK/STAT3), the mitogen-activated protein kinase/extracellular regulator kinase (MAPK/ERK), the c-Jun N-terminal Kinase (JNK), and the phosphatidylinositol-3-kinase/protein kinase B (PI3K/AKT) pathways (**Figure 1C**) (19–21). To a lesser extent OSM signaling may also activate additional STAT proteins, including STAT1 and STAT5, depending on the cell type (22, 23). OSM is synthesized and secreted by a variety of cells; primarily activated macrophages, monocytes, T cells, dendritic cells, and neutrophils (1, 2, 24). OSM acts in a pleiotropic fashion, contributing towards a variety of physiological functions such as hematopoiesis, stem cell differentiation, liver regeneration, and inflammation. While some of these effects are similar to other IL-6 family members, many are unique (5, 11, 15). Over the course of the last 30 years OSM has been demonstrated to play a significant role in a variety of processes and diseases, yet successful development of an anti-OSM clinical therapeutic has not yet reached FDA approval despite mounting evidence that such a therapeutic is necessary for a multitude of diseases. In this review, we will describe: i) the different roles that OSM plays within the human body regarding its function in inflammatory diseases; ii) the role of OSM in multiple cancer types; and iii) a detailed analysis of current targeted therapies designed to disrupt OSM signaling.

## 2 OSM in diseases

A significant difficulty with developing treatment strategies to block OSM stems from the pleiotropic nature of the cytokine. As indicated in **Table 1**, OSM has a variety of positive and negative effects on several diseases in the body. Systemic inhibition of OSM signaling in the body for extended periods of time may be beneficial with respect to some diseases, but detrimental in cases that rely on the proinflammatory response for healing. This section will describe the role OSM plays in a variety of diseases.

**Table 1:** OSM in Diseases.

Disease	Type	Impact of OSM	References
Diseases associated with OSM signaling			
Arthritis	Rheumatoid and Osteoarthritis	ECM turnover, cartilage degradation, osteoblast proliferation/differentiation	(23, 25–32)
Bone	Neurogenic Heterotopic Ossification	Osteoclast/osteoblast imbalance	(33–37)
Inflammatory Bowel Disease	Chron’s Disease, Ulcerative Colitis	Presence in intestinal mucosal cells signify anti-TNF therapy resistance	(38–45)
Lung Diseases	Pulmonary Fibrosis, Asthma	ECM and Pro-fibrotic macrophage accumulation, airway remodeling	(46–50)
Cutaneous Inflammatory Diseases	Psoriasis, Atopic Dermatitis	Increase in keratinocyte proliferation and differentiation	(51–57)
Oral Disease	Gingivitis, Periodontal Disease	Increase presence and activation of Th1 cells	(58–62)
Liver Diseases	Fibrosis, Cirrhosis	Increased TIMP-1 expression, reduction in fibrinolysis, increase in myofibroblasts	(63–72)
Central Nervous System Disorders	HIV-1 Associated Neurocognitive Disorders, Alzheimer’s	Inhibits glutamate uptake, BBB impairment	(73–77)
Heart	Atherosclerosis	Proinflammatory response in smooth muscle cells	(78–86)
COVID-19	Cytokine Storm	Present in Cytokine Storm associated with severe COVID-19 infection.	(87–91)
Cancer	Many	Tumor cell detachment, invasion, metastasis	See **Table 2**
Normal conditions associated with OSM signaling			
Liver	Regeneration, development, acute injury	Hepatocyte differentiation, proliferation, tissue remodeling	(63–72)
Bone	Bone homeostasis, fracture repair	Osteoblast differentiation/proliferation	(33–37)
Central Nervous System	Multiple Sclerosis, Spinal Cord Injury	Increase TIMP-1 and MCL-1 expression, remyelination, neuroprotective effects	(73–77)
Heart	Post myocardial infarction	Increase in angiogenesis, dedifferentiation	(78–86)
Wound Healing	Early Wound Healing, Scar Formation	Neutrophil recruitment, excessive scar prevention	(92–95)
Pregnancy	Placental development, Trophoblast invasion	Increase MMP2/9, regulation of HCG	(96–98)

### 2.1 Arthritis

Arthritis is a disease referring to chronic inflammation in one or more joints of the body, with the two most common and studied types being: rheumatoid arthritis and osteoarthritis. Rheumatoid arthritis (RA) is a chronic autoimmune disease involving inflammation of the lining of joints. It has been shown that the presence of OSM and another proinflammatory cytokine, interleukin-1β (IL-1β), play an important role in development of the rheumatoid joint (25, 26). In multi-cell culture systems, OSM-mediated signaling has been identified as an initiator of extracellular matrix (ECM) turnover as well as human cartilage degradation; potentially though the secretion of matrix metalloproteinase (MMP) -1 and -13 (23). Through regulation of various proinflammatory mediators, OSM in rheumatoid arthritis synovial fibroblasts (RAFLS), increases the detrimental effects of tumor necrosis factor alpha (TNFα) via activation of STAT3 signaling (27). Inhibition of JAK/STAT signaling with tofacitinib (a JAK inhibitor) resolved inflammation through metabolic reprograming of biopsied RALFS (28). *In vivo* studies demonstrated that both collagen-induced and pristane-induced arthritis mouse models, showed significant improvement in severity (p<0.01) and number of affected paws (p<0.01) when administered an anti-OSM antibody (29). In response to the strong association of OSM with RA, two clinical therapeutics have entered clinical trials (GSK315234 and GSK2330811) discussed in further detail below (see Anti-OSM Therapeutics).

The effects of OSM are not limited to rheumatoid arthritis, as studies have shown its effects in the development of the much more prevalent osteoarthritis (OA). OA is characterized by articular cartilage destruction and an inflammatory response due to mechanical wear on joints (30). *In vitro* analysis in primary OA osteoblasts has demonstrated that degradation and inflammation could be due to endothelin-1 (ET-1) trans-activating OSM via Ets-1 (31). Increased levels of OSM in synovial tissues induce bone formation through osteoblast proliferation and differentiation following cartilage degradation by inhibition of Notch signaling (32). Currently no clinical trials have evaluated the efficacy of anti-OSM therapeutics for OA.

### 2.2 Bone

OSM signaling regarding bone tissue is complicated, as there is evidence to suggest benefit and harm from its activation. It is well known that OSM induces differentiation of mesenchymal stem cells (MSCs) into osteoblasts though the STAT3 signaling pathway (17, 33, 34). This can be beneficial in respect to new bone formation in osteoporosis and arthritis as well as remodeling of bone during fracture repair (33). However, there is significant research suggesting that OSM has an overall negative effect in respect to bone. While osteogenesis is being stimulated, the anabolic signal suppresses the reabsorption signal-controlled receptor activator of nuclear factor-kappa beta ligands (RANKL) signaling, repressing osteoblast-mediated osteoclast differentiation in bones (34). OSM-mediated STAT3 signaling has also been associated with the development of neurogenic heterotopic ossifications, which is the formation of bone tissue in periarticular muscles, an incapacitating complication of traumatic brain and spinal cord injuries (35). Other studies have indicated that STAT3 signaling through OSM is necessary for osteoclast formation and subsequent bone resorption (36, 37). It is clear that OSM plays a role in bone remodeling, however there is evidence suggesting it promotes the differentiation of both osteoblasts and osteoclasts, leading to some potential questions about the true effects of OSM signaling among bones.

### 2.3 Inflammatory bowel disease

Inflammatory bowel disease (IBD) is a collective term describing disorders that involve chronic inflammation of the small intestine. The two main forms of IBD are ulcerative colitis (UC) and Chron’s disease (CD) (38). It has been demonstrated that OSM and OSMR are overexpressed in many IBD lesions (39). While anti-tumor necrosis factor alpha (TNFα) antibodies such as infliximab, adalimumab, certolizumab, and golimumab have long been available as treatment strategies for CD and UC, they are far from perfect. Approximately one third of patients do not respond to infliximab initially, and between 23% and 46% develop resistance to anti-TNFα therapies within 12 months of treatment (40, 41). A recent retrospective cohort study demonstrates that patients with increased levels of OSM had a lower chance of remaining in remission 1 year after starting anti-TNFα therapies (42). There are other IBD treatment options available, some of the more viable options include JAK/STAT inhibitors, indicating OSM inhibition upstream may be beneficial (43). In a study analyzing over 200 patients with IBD, those with high OSM and OSMR expression before treatment with infliximab demonstrated reduced efficacy of the therapy from 69-85% to just 10-15% (39). *In vivo* IBD mouse studies comparing wildtype and *Osm-/-* showed that lack of OSM signaling led to a decrease in overall pathology (p<0.0001), leukocyte infiltration (p<0.0001), epithelial and goblet cell disruption (p<0.0001), area affected (p<0.0001), and severity of disease features (p<0.0005) (39). Additionally, OSM has been shown to mediate STAT3-dependent upregulation of serin protease inhibitors (SERPINS), which have anti-apoptotic effects in intestinal epithelial cells that lead to inflammation and a disrupted intestinal epithelial barrier (44). Recent studies have also shown that the use of berberine, an isoquinoline alkaloid used to treat chronic UC, interferes with the production of OSM from T cells, neutrophils, dendritic cells, and macrophages, as well as inhibiting OSM activation of stromal cells and recruitment of immune cells (45). As seen in other diseases with inflammation as a hallmark, OSM signaling is a key component in disease pathogenesis, and in this case, it also appears to interfere with pharmacological treatments.

### 2.4 Lung diseases

OSM plays an important role in the development and progression of pulmonary fibrosis and chronic lung inflammatory diseases such as asthma. OSM levels are upregulated in patients with pulmonary fibrosis, due to increased lung inflammation as well as accumulation of ECM proteins (46). Evidence suggests that an increase in OSM and IL-6 leads to the accumulation of profibrotic macrophages, in turn increasing bleomycin-induced lung fibrosis (47). Chronic inflammatory diseases of the lung such as asthma also showed a significant increase in OSM expression (48). In severe asthma, poor disease prognosis is characterized by an increase in number of epithelial mucus producing cells, peribronchial fibrosis, and smooth muscle contractility through follistatin-like 1 induction of OSM expression (49). Recent studies have also demonstrated that overexpression of OSM leads to an increase in resistin-like molecule alpha in airway epithelial cells, leading to rearrangement of the ECM in mouse lungs (50). The increase in OSM signaling in the lungs with respect to these chronic inflammatory diseases demonstrates its potential to be used as a therapeutic target.

### 2.5 Cutaneous inflammatory diseases

Being the human body’s largest organ, and our first line of defense, the skin plays an important role in inflammatory processes and responses. Diseases such as psoriasis and atopic dermatitis are chronic inflammatory skin diseases that affect over 7.5 million individuals within the United States and are characterized by keratinocyte hyperplasia, proliferation, and altered differentiation (51, 52). These diseases demonstrate an increase in skin infiltrating T lymphocytes that lead to secretion of OSM and an increase in OSMRβ on keratinocytes, increasing keratinocyte activation through STAT3 signaling (52). When OSM is overexpressed in the skin, its proinflammatory effects have been demonstrated both *in vitro* and *in vivo* (53). Studies conducted in mice show that intradermal injection of OSM, but not IL-6, regulates the expression of genes responsible for skin inflammation and epidermal differentiation, including S100A8/9, cytokeratin-10, filaggrin, and a number of other cytokines (52). Additionally, OSM has recently been identified as a significant contributor to chronic pruritus (itching). *Tsen and Hoon et al.* discovered that OSMRβ is preferentially expressed by itch-selective sensory neurons and that OSM can directly modulate itch-selective neurons during chronic skin inflammation (54). Functional studies showed that OSM acts in a unique fashion compared to other pruritogens, being that OSM causes potentiation of neural response to pruritogens and that OSM increases sensitization of sensory neurons, resulting in tonic action potential firing of itch-selective neurons. Another study showed that OSM and IL-31 stimulate dermal cells expressing IL-31RA and OSMRβ, which may further promote itch and inflammation in patients with prurigo nodularis, a chronic skin dermatosis (55).

Scleroderma is an inflammatory autoimmune disease that is characterized by increased activation of fibroblasts leading to accumulation of connective tissue that results in chronic inflammation on the surface on the skin and internal organs (56). Elevated OSMRβ levels have been found in fibroblasts as well as dermal endothelial cell of scleroderma patients. *In vitro* of human dermal microvascular endothelial cells treated with OSM and IL-6 displayed induced cell migration and proliferation, as well as stimulation of proinflammatory genes, and genes associated with endothelial to mesenchymal transition including STAT3, ET-1, zinc finger protein SNAI1 (SNAIL1), transforming growth factor-β 3 (TGFβ3), and its receptor TGFβ3R (57). There is currently a clinical trial for a monoclonal antibody against OSM, in patients with systemic scleroderma, which will be discussed in greater detail in Anti-OSM therapeutics.

### 2.6 Oral diseases

OSM and other cytokines play an important role in the progression of periodontal disease, a gum infection typically caused by poor brushing and flossing habits. In studies investigating levels of OSM in patients’ gingival crevicular fluid, OSM concentrations increase as disease severity progresses from early-stage disease to chronic periodontitis (58–60). Another study focused on T-helper type 1 (Th1) cells, which have detrimental effects in regard to periodontal disease through the stimulation of alveolar bone loss (61). OSM and IL-1β together increased the expression of chemokine (C-X-C motif) ligand 10 (CXCL10), a chemokine related to Th1 cell migration, as well as intracellular adhesion molecule 1 (ICAM-1) that is important in retention and activation of Th1 cells in inflamed tissue (62). CXCL10 and ICAM-1 expression were both suppressed when inhibitors of NF-κB and STAT3 were introduced, indicating that OSM-mediates STAT3 signaling and IL-1β-mediated NF-κB signaling may promote infiltration and retention of Th1 cells, leading to periodontal disease (62). Overall OSM signaling continues to demonstrate negative downstream effects through activation of several different signaling molecules.

### 2.7 Liver

Inflammation is a key driver in liver disease, and it has been demonstrated that OSM plays a profibrogenic role in the progression of chronic liver disease (63–65). Administration of OSM to human hepatocellular HepG2 cells in culture increased expression of GP73 (a glycoprotein biomarker for cirrhosis and hepatic cell carcinoma), indicative of the effects OSM has on promoting chronic liver disease progression (63). OSM’s role in fibrosis is characterized by promoting the expression of tissue inhibitor of metalloproteinase 1 (TIMP-1), which both suppresses fibrinolysis in hepatic stellate cells (HSCs) as well as promotes fibrogenesis through induction of Type 1 collagen expression (64). Recent research also suggests that OSM promotes fibrosis in non-alcoholic fatty acid liver disease (NAFLD) through stimulating migration of hepatic myofibroblasts (MFs) that originate from HSCs (65). A recent study analyzed serum levels of 83 patients with NAFLD and non-alcoholic steatohepatitis (NASH) who also have hepatocellular carcinoma (HCC) and found that the presence of HCC further increased OSM concentrations (66). It has also been found in chronic hepatitis C that antigen presenting cells release OSM following interaction with CD40L present on active CD4+ T cells (67). This OSM does not result in a large notable effect, as OSMRβ and LIFR expression is also downregulated both *in vitro* and in patients with chronic hepatitis C; however, the increase in ligand is thought to be noteworthy (67).

In addition to affecting liver fibrosis and progression of chronic liver disease, OSM also plays an important role in liver regeneration (64, 68–71). Following acute liver injury, it is primarily oval cells that are responsible for liver regeneration (72). More research has shown that OSM is able to induce the differentiation of these oval cells into hepatocytes *in vitro* (69). *In vivo* studies demonstrated that OSMRβ knockout mice had impaired hepatocyte proliferation and tissue remodeling following induced liver injury, indicating its importance in regeneration (71). Furthermore, administration of OSM in wild type mice mitigated liver injury through prevention of apoptosis and tissue destruction (71). It was also demonstrated that OSM gene therapy in rats effectively increases proliferation and the anti-apoptotic effects of on hepatocytes, leading to liver regeneration (70). Through the upregulation of hypoxia-inducible factory 1 α (HIF1α) and HIF1 transcription, OSM demonstrates its importance as an upstream mediator of vascular endothelial growth factor (VEGF) and plasminogen activator inhibitor 1 (PAI1), both of which are important for angiogenesis and tissue remodeling respectively (68). While OSM does appear to play an important role in regeneration following acute liver injuries, continuous activation of OSM has a very different effect with respect to fibrosis and chronic liver disease suggesting its prolonged presence may be harmful.

### 2.8 Central nervous system

In diseases related to the central nervous system, research has indicated that OSM plays various roles with both detrimental and beneficial outcomes. Studies have been conducted displaying various negative effects that OSM has on the central nervous system. Diseases such as HIV-1-associated neurocognitive disorders and Alzheimer’s disease have displayed elevated levels of OSM (73). Specifically, through JAK/STAT3 signaling, OSM inhibits glutamate uptake in astrocytes resulting in neuronal excitotoxicity (73). OSM also induces blood brain barrier impairment in mice, through prolonged STAT3 signaling in pericytes (74). As discussed previously, STAT3 activation initiated through OSM signaling *in vivo* has also been shown to increase neurogenic heterotrophic ossifications in damaged muscles following spinal cord injuries (35).

In diseases characterized by the loss of myelination such as multiple sclerosis, overexpression of OSM has been shown to mediate the expression of tissue inhibitor of TIMP-1, promoting a beneficial remyelination (75). Following mild and severe spinal cord injuries, elevated OSM signaling results in improved recovery and neuroprotective effects by promoting neurite outgrowth, increasing serotonergic fiber plasticity, and protecting primary neurons from cell death (76). OSM also stimulates the expression of myeloid cell lukemia-1 (MCL-1), in turn enhancing mitochondrial bioenergetics and increasing neuroprotective effects against 3-nitropropionic acid in cortical neurons (77). The pleotropic nature of OSM is clearly demonstrated with respect to the central nervous system, especially concerning the inflammatory response following spinal cord injuries.

### 2.9 Heart

When it comes to function of the heart, research has indicated that OSM plays various roles with both detrimental and beneficial outcomes. Regarding atherosclerosis development and progression, the vast majority of research suggests that prolonged STAT3 activation through OSM signaling has a negative impact on arterial vessels, which leads to atherogenesis (78–81). However, one recent study showed that chronic OSM administration in mice reduced atherosclerosis development, and patients with higher levels of serum OSM had improved coronary heart disease survival probability (82). This differing research continues to cloud the role OSM plays in the heart.

Cardiomyocytes are the workhorse of the heart, and OSM plays a key role in the dedifferentiation of cardiomyocytes (83, 84). This dedifferentiation leads to protective effects during and following acute myocardial infarction (MI) (83–85). Knockout of OSM signaling following MI in mouse models suppressed cardiomyocyte dedifferentiation, resulting in decreased heart function, while OSM treatment induced remodeling, stem cell marker expression, and improved cardiac function (84). Inhibition of OSM treatment reduced cardiomyocyte function following MI, however, improved performance in dilated cardiomyopathy (DCM) indicates negative effects of OSM long-term (84). Other studies done in mice have shown that OSM increases cardiac function following MI through the inhibition of apoptosis and fibrosis, while stimulating angiogenesis. OSM treated mice had significantly increased capillary density as well as increased expression of pAKT and the angiogenic factors, VEGF and basic fibroblast growth factor (bFGF) (85). More *in vivo* studies have indicated that OSM alleviates post MI dysfunction by enhancing cardiomyocyte autophagy through the inhibition of mammalian Ste20-like kinase 1 (Mst1) (86). Activation of Mst1 has been shown to cause (DCM) as well as inhibit cardiomyocyte autophagy (86). OSM signaling is a crucial component to the heart’s response to acute stressors, but when its presence is prolonged, it can have other effects.

### 2.10 Wound healing

It is well known that following almost any cut or abrasion an inflammatory response is triggered. OSM has been shown to be an important player in the early stages of the wound healing process under normal and diabetic-impaired healing conditions *in vivo* (92). This increase in OSM at the site of inflammation has been tied to the early influx of polymorphonuclear neutrophils into the wound site, but if OSM is around for too long it can actually impair the healing process in chronic diabetic wound conditions (92). Other studies have demonstrated the role OSM has in the scarring process (93, 94). Hypertrophic and keloid scars are both abnormal wound responses to trauma, inflammation, surgery, or burns. Keloid scars are typically considered worse than hypertrophic scars as keloid scars often increase in size, can develop months after surgery, and fail to improve appearance over time even with surgical intervention (93). Increased levels of OSM have been found in hypertrophic scars but not keloid scars, and it has been demonstrated that the increase in OSM served as protection against excessive scarring through suppression of TGFβ1-induced ECM protein expression (94). Other studies have shown similar benefits that OSM has in respect to late and early wound healing, differentially demonstrating an anti-inflammatory effect (95). It again seems that in the case of wound healing and scarring, more research is needed to clarify the contributions of OSM.

### 2.11 Pregnancy

A lot of changes take place in a person’s body during pregnancy. Studies done in humans have shown OSM is present in high concentrations in pregnant women when compared to non-pregnant women, as well as in placental tissue in all three trimesters (96). OSM is especially relevant in the early stages of pregnancy through the upregulation of human chorionic gonadotropin, demonstrating importance in placental endocrine function (96). Through STAT3 and ERK1/2 signaling, OSM and LIF are responsible for trophoblast invasion and placental development, both important steps in the early stages of pregnancy (97). *In vitro* studies show trophoblast invasion is induced through the upregulation of matrix metalloprotease 2 (MMP2) and MMP9 by both OSM and LIF, either synergistically or separately, in some but not all cell types used (97). Other studies also suggest that OSM increases protein expression and enzymatic activity of MMP2 and MMP9, leading to the invasion of primary trophoblasts through STAT3 signaling under hypoxic conditions typically found during trophoblast invasion in early pregnancy (98). While OSM presence is increased during pregnancy and is a known STAT3 activator, there is more research suggesting LIF signaling is primarily responsible during early pregnancy.

### 2.12 Cytokine storm and COVID-19

The coronavirus disease 19 (COVID-19) is caused by the novel severe acute respiratory syndrome coronavirus 2 (SARS-CoV-2) and has sparked a global pandemic since its introduction in humans in late 2019. It is well known that cytokines play an important role in developing an innate immune response during viral infection. There has been evidence suggesting that cytokine storms, characterized by an excessive and dysregulated immune response, play a significant role in pathogenesis of SARS-Cov-2 infection (87–89). Retrospective research studies conducted around the world have shown that a hyperinflammatory state, indicated by the presence of IL-6, IL-10, and TNF-α, is a significant predictor of mortality (89). A different study found increased OSM along with a number of other inflammatory proteins present in lung and spleen tissue in 13 postpartum subjects with fatal COVID-19 infections (90). Other retrospective studies done in Hong Kong and Atlanta Georgia have demonstrated an increase in cytokines and proinflammatory mediators such as IL-6, TNSF14, EN-RAGE, and OSM are correlated with disease severity (91). While there is a lot to be said about the role of cytokines in COVID-19, OSM’s role has yet to be fully elucidated.

## 3 OSM in cancer

As previously stated, the pleiotropic nature of OSM causes it to exert differing effects on various cell types. While OSM has been investigated in a multitude of diseases, a particular area of interest is cancer biology. In the tumor environment, OSM often acts in a deleterious fashion through multiple different mechanisms, though it is noted that OSM can have a positive effect on specific cancer types, making it a particularly interesting cytokine to study (99–101). In this section, we will provide a thorough analysis of the role OSM plays in a multitude of cancers. **Table 2** provides a list of the cancers that will be discussed in the following section.

**Table 2:** OSM in Cancer.

Location	Cancer Type	Impact of OSM	Reference
Pro-tumorigenic Effects of OSM			
Breast	Ductal Carcinoma	Overexpression linked with poor prognosis and creates a more CSC phenotype; Increases EMT, motility, invasion, and metastasis; Recruits neutrophils and surrounding tissue to express OSM	(20, 102–135)
Cervical	Squamous Cell Carcinoma	OSMRβ overexpression leading to EMT and increased skeletal metastasis	(135–143)
Ovarian	Epithelial Carcinoma	Auto/paracrine signaling loop in malignant OC; Increased proliferation, and metastasis dependent on STAT3 Increase in keratinocyte proliferation and differentiation	(144–148)
Prostate	Ductal Adenocarcinoma	Increased VEGF and u-PA expression and induced EMT in prostate epithelial cells	(149–155)
Testicular	Leydig Cell Carcinoma	Upregulation of OSM in functioning neoplasms	(156, 157)
Colon	Adenocarcinoma	More advanced and aggressive CRC have higher OSM serum level and lower survival; OSMRβ polymorphisms	(158–163)
Gastrointestinal	Adenocarcinoma	Differential expression of OSM in the grades of GI cancers could be used as biomarker.	(164–169)
Pancreatic	Ductal Adenocarcinoma	Overexpressed OSM in the serum, causes EMT, and greater metastasis to the lung *in vivo* dependent on STAT3	(170–175)
Bladder	Urothelial	Genetic mutations can cause an overexpression of the OSMRβ, leading to increased signaling	(176–178)
Lung	Adenocarcinoma	Induce EMT, increase fibroblast activation, OSMRβ overexpression is correlated to poor prognosis	(179–187)
Brain	Astroglioma, astrocytoma, adenoma, glioblastoma, glioma, medulloblastoma, meningioma	Three-fold increase in VEGF, seven-fold when in conjunction with IL-1β	(52, 182–184, 188–198)
Squamous Cell Carcinoma	Cutaneous & Oral Squamous Cell Carcinoma	Promotes proliferation, migration, and inflammation *in vitro* and *in vivo*	(199–210)
Kaposi’s Sarcoma	Sarcoma	Mitogen and autocrine growth factor, promoter of bFGF	(4, 136, 211–217)
Misc. Sarcomas	Osteosarcoma, Chondrosarcoma, Ewing Sarcoma	Increased MMP2, VEGF, and proliferation	(218–227)
Melanoma	Melanoma	Antigen-silencing, resistance to inhibitory OSM singling in > Stage 3 patients	(225, 228–240)
Anti-tumorigenic Effects of OSM			
Multiple	Chondrosarcoma	Cell cycle arrest through JAK3/STAT1 signaling, decreased proliferation and enhanced apoptosis	(225)
Skin	Melanoma	Activates STAT5B and MAPK inhibiting proliferation; Increased SOCS3 with decreased OSMRβ expression	(225, 228–240)

### 3.1 Breast

Breast cancer is the most common cancer among women, and the second leading cause of cancer-related deaths in the United States. While OSM has historically been identified as an inhibitor of breast cancer proliferation (15, 102, 103), overexpression of OSM and OSMRβ has been linked to decreased overall survival, decreased reoccurrence-free survival and decreased metastasis free survival in breast cancer patients (104–107). Immunohistochemical analysis in benign human breast lesions have shown low expression of OSMRβ (11.7%) and gp130 (23.5%) proteins. However, in infiltrating carcinomas; high expression of OSMRβ (77.5%) and gp130 (74.1%) proteins have been seen, with OSM localized in 100% of tumor samples studied (107). At the molecular level, OSM via STAT3 signaling has been shown to inhibit c-Myc expression in human mammary epithelial (HMEC) cells, but constitutively overexpressing c-Myc HMEC cells gain the capacity for anchorage-independent growth in the presence of OSM-mediated PI3K-AKT signaling, suggesting c-Myc acts as a molecular switch to alter response of mammary epithelial cells (108). OSM has also been shown to promote a cancer stem cell (CSC)-like phenotype and pro-survival phenotype for breast cancer cells (36, 109). It can also create a pre-metastatic environment in bone by inducing osteoclast differentiation, increasing the possibility of bone metastases for breast cancer cells expressing a high level of OSM suggesting that the role of OSM in breast cancer is not tumor proliferation, but rather migration and invasion (36, 110, 111).

Examining patient tissue using microarray analysis, OSM expression was revealed to be the highest in patients with ductal carcinoma *in situ* (DCIS) (109). This highlights the possible role that OSM could have in progressing early grade tumors. Paracrine and autocrine signaling of OSM has been shown *in vivo* to increase the amount of circulating tumor cells (CTC), epithelial to mesenchymal transition (EMT), as well as increased metastasis to lungs and decreased survival (109). OSM can also induce CD44 ^high^/CD24^low^ phenotype allowing OSM to promote a CSC- like property, as well as increase detachment and migration of ER+ cells, while EMT remains independent of CD44 induction (112).

Recently, there has been increasing evidence that OSM operates differently in the varying subtypes of breast cancer (104, 106). At diagnosis, breast cancer patients are categorized into different subtypes based on expression of three receptors: estrogen receptor-alpha (ERα), progesterone receptor (PR), and human epidermal growth factor receptor 2 (HER2) (113–120). ERα status is important for clinical management of breast cancer since tumor cells that are ERα+ are usually less aggressive and can be treated with endocrine therapies (121). OSM has shown the ability to downregulate the expression of ERα, which in turn increases the OSM signaling cascade and migratory effects that its pathways have *in vitro* (105). *In vivo*, high OSM expression was correlated with decreased ERα (p < 0.01) and PR (p < 0.05) expression, and a shorter reoccurrence-free survival (p < 0.0001) (105). OSM has also been shown to promote secretion of IL-6 in ERα- cells and not in ERα+ cells, further suggesting that OSM plays unique roles in ERα+ versus ERα- breast cancer (104, 106). This illustrates that OSM can increase the metastatic potential of breast cancer cells as well as make them more difficult to treat in a clinical setting.

Triple negative breast cancers (TNBCs) are highly aggressive, metastatic, and therapeutically difficult to treat due to their lack of, or low expression of receptors commonly targeted for therapeutics (122, 123). It has been shown that patients with TNBC and high OSM expression have a greater abundance of cells with a cancer stem cell (CSC) phenotype due to OSM/STAT3/SMAD3 signaling, which promotes growth of the tumor and leads to poor clinical outcomes for patients (111, 124). Similar research also evaluated OSM-mediated MEK/ERK signaling and found that blocking ERK abolished the growth inhibition characterized by OSM, but only in triple-negative MDA-MB-231 cells (21). Interestingly, interferon-β (IFN-β) can repress this OSM-mediated tumor initiation and CSC phenotype, but mRNA of endogenous IFN-β is repressed directly by OSM. IFN-β is suggested as a possible therapeutic to OSM in TNBCs, following a more comprehensive investigation of the relationship between these two cytokines (125). OSM can also perpetuate a chronic inflammatory environment that is detrimental to the prognosis of breast cancer patients due to the recruitment and/or induction of other inflammatory cytokines that are known to promote a metastatic phenotype in breast cancer. Induction of IL-6 is directly caused by the synergistic effects of both OSM and IL-1β (106). Analysis of The Cancer Genome Atlas (TCGA) breast cancer dataset shows that these three cytokines in high concentrations lower patient’s survival rate (p < 2.2x10^-23^) (106).

The tumor microenvironment (TME) plays a large role in the progression of breast cancer, through the complex interactions of tumor cell-to-cell communications, secretions of infiltrating immune cells, and communications of surrounding tissues (126–129). OSM has been shown to directly bind to extracellular matrix proteins, which can protect it from proteases and preserve bioactive accumulation of OSM near within the TME for long periods of time (130). Stromal OSM production has also been recently shown to play a significant role in breast cancer progression by reprogramming fibroblasts within the TME towards a more tumorigenic phenotype and increase proinflammatory myeloid cell recruitment (131). OSM signaling also leads to TME remodeling in breast cancer as OSM induces the expression of lysyl-oxidase like 2 (LOXL2), which leads to crosslinking and alignment of collagen I fibers present in the stromal ECM (132). Presence of OSM within the breast tumor microenvironment has been shown to be provided by tumor associated neutrophils (TAN’s), tumor associated macrophages (TAM’s), and monocytes. Stromal OSM/OSMRβ has recently been shown to play a distinct role in breast cancer progression (131). TAN’s co-cultured with human breast cancer cell lines have also been shown to increase TAN-mediated secretion of OSM throughout the tumor microenvironment, which in turn leads to increased secretion of the pro-angiogenic factor VEGF from human breast cancer cells (133). Neutrophils co-cultured with human breast cancer cells, MDA-MB-231 and T47D, also increased the number of viable cells that underwent detachment and increased invasive capacity *in vitro*, as measured by cell-cell/cell-substratum detachment and Matrigel invasion assays (133). This suggests that TANs can increase the expression of OSM at the site of the tumor and promote angiogenesis and metastasis of breast cancer cells.

Adipose tissue has also been shown to play a role in the progression of breast cancer, with obese post-menopausal women having twice as high of a mortality rate as compared to low body-mass-index post-menopausal women (134). Breast cancer-associated adipose tissue from patient tumors display high secretion of OSM, alluding to the paracrine signaling that could initiate a metastatic phenotype in breast cancer cells (20). When co-cultured with breast cancer cells, the adipose tissue induced EMT and increased the invasiveness of the breast cancer cells in a STAT3 dependent manner (20). Another direct target for OSM/STAT3 signaling is fascin, an actin-bundling protein that localizes to filopodia and functions in cell-to-cell interactions and cellular motility. STAT3 can directly bind to the promoter region of the fascin gene to upregulate its expression to increase cellular migration (241).

All of this evidence collectively highlights the role OSM has in breast cancer progression and metastasis. As metastatic breast cancer has the poorest survival rate at 29% (135), developing a therapeutic to inhibit OSM may dramatically prolong the life of patients and lead to better survival outcomes.

### 3.2 Cervical cancer

Cervical carcinoma ranks as the second most common cause of cancer deaths among women, with approximately 270,000 deaths per year globally (135). A vast majority of cervical carcinomas are squamous cell carcinomas (SCC) that arise from precursor squamous intraepithelial lesions (199). In 2007, *Ng et al* evaluated potential genes showing high-frequency copy number-driven changes in expression in cervical SCC and discovered that the OSMR gene was significantly higher in cervical SCC cases when compared to patients with precursor cervical squamous intraepithelial legions and gain of OSMR was significantly associated with adverse overall patient survival, (p=0.046) and may increase radio-resistance in cervical SCC (242). Additional work by this group directly examined the consequences of *OSMR* overexpression in vitro and discovered that OSM signaling dramatically increases cell migration, invasion, and induction of tumorigenic factors such as IL-6, HIF2-α, VEGF, and transglutamase 2 (TGM2) a calcium-dependent crosslinking enzyme that catalyzes post-translational protein modifications, yet no evidence of OSMR overexpression improving radio-resistance was found (243–245). A separate group investigated the sensitivity of cisplatin therapy of cervical SCC cells and found that while STAT3 phosphorylation dramatically increased in pre-cancerous cervical cancer legions, it declined when comparing to cervical SCC, and cervical SCC cells pre-treated with OSM were more responsive toward cisplatin-based chemoradiotherapy, via upregulation of STAT3-mediated interferon-regulatory factor 1 (IRF1) expression (246, 247). More recent work has also evaluated *OSMR* overexpression utilizing clinical data from the TCGA CESC (cervical cancer) database and found that patients with high OSMR expression display increased expression of mesenchymal makers such as SNAI1, SNAI2 and zinc finger E-box-binding homeobox (ZEB1) (248). Additionally, using 3D culture models and mouse *in vivo* models and found that OSMRβ-overexpressing cervical SCC cells exhibit increased EMT, stem cell-like properties as well as increased lung colonization and skeletal metastases *in vivo* (248). These studies together suggest that cervical cancer cells with increased OSM signaling and OSMRβ overexpression are more aggressive, and lead to worse overall survival in cervical SCC patients. While few studies have implicated a possible radioresistant role for high OSMRβ patients, it has not been fully evaluated. Nonetheless, OSMRβ overexpression may be a potential clinical marker for cervical cancer patients, HER2 in breast cancer, and an anti-OSMRβ monoclonal antibody could improve outcome for patients with cervical cancer.

### 3.3 Ovarian

Ovarian cancer is the fifth most common cancer in woman and the leading cause of death among gynecological cancers (135). While IL-6 family cytokine members have been evaluated in the progression of ovarian cancer, OSM has not been extensively studied (136). A small study consisting of 29 malignant ovarian carcinoma patients revealed OSM was expressed in all 29 primary malignant ovarian carcinomas (MOC). Additionally, the same group analyzed 25 primary ovarian carcinomas samples (OC) for LIFRβ and OSMRβ expression and found all 25 primary OC samples expressed LIFRβ and 14 out of 25 expressed OSMRβ (137). Overexpression of both LIFRβ and OSMRβ in turn has been shown to constitutively activate STAT3 nuclear signaling in 74% of MOC’s tested, suggesting OSM signaling is frequently present in malignant ovarian carcinoma (137). *Li et al.* further supported this work, showing that OSM treatment enhanced proliferation of OC cells *in vitro* in a STAT3 dependent manner (138). Interestingly, in contrast to these studies, a group evaluating 239 epithelial ovarian cancer patients (19 with low stage and 220 with high stage) and 169 controls identified that OSM was significantly downregulated (-2.62-fold change in early stage and -2.65 in late stage) in the leukocytes fraction of ovarian cancer patients compared to healthy patients (139). More recent research however has further evaluated the role of OSM in ovarian cancer and found that *OSMR* is highly expressed in ovarian cancer cells, cancer associated fibroblasts, and endothelial cells of patient samples, and is highly expressed when compared to normal adjacent tissues. Additionally, this study showed human ovarian cancer cell lines overexpressing OSMRβ were found to promote colony formation, migration, invasion, and spheroid-forming capabilities, and that an anti-OSMRβ monoclonal antibody reduced the growth and peritoneal spread of ovarian cancer cells using a mouse *in vivo* ovarian cancer model (140). From this evidence, while the presence of OSM in ovarian cancer patients is not confounded, OSM-mediated STAT3 signaling does impact ovarian cancer progression. It could be hypothesized that similarly to cervical cancer, OSMRβ rather than OSM is the more favorable target against ovarian cancer metastasis and could be used as a clinical marker for disease progression and patient outcomes.

### 3.4 Prostate

Prostate cancer (PC) is both the second most common cancer and second leading cause of cancer related deaths in men. PC is one of the four most common cancer types, and with reduction rates within the population plateauing, there is a need to better understand the mechanisms that PC utilizes to persistently remain present within the population (141).

OSM treatment on prostate carcinoma cells (DU145) *in vitro* has been shown to increase the amount of urokinase-type plasminogen activator (u-PA), a serine protease that degrades ECM proteins leading to increased invasion and metastasis *in vitro*, as well as VEGF measured by means of ELISA (142). This correlation was also seen clinically evaluating 47 male patients: 20 with benign prostatic hyperplasia (BPH), 20 with non-metastatic PC, and 7 with metastatic PC. Patients with metastatic PC displayed a significant increase in plasma levels of IL-6 (p<0.0001), OSM (p<0.009), VEGF (p<0.016), and u-PA (p<0.0001) compared to the other disorders (142). Interestingly, OSM was also shown to induce tumorigenic properties, including EMT progression and migration of non-transformed human prostate epithelial cells via STAT3 signaling (143). A separate study also highlighted miR-181b-5p as a potential inhibitor of OSM-mediated prostate cancer progression using *in vitro* mouse prostate cancer cell lines. In the presence of OSM, miR-181b-5p was shown to inhibit proliferation, invasion and metastasis of mouse cell lines, while also repressing the levels of osteoclastogenic factors such as IL-6, AREG, and OPG that could prevent prostate cancer metastasis to bone (144).

PC commonly starts as an androgen-dependent tumor, making androgen-depriving therapeutics a useful first-round treatment strategy; however, 20-30% of patients exhibit recurrence of PC with a more aggressive androgen-negative phenotype that is difficult to treat (142, 145). Recent research has examined how exercise affects patients with advanced prostate cancer or patients receiving androgen deprivation therapy and have noticed elevated levels of OSM (146, 147). The implications of this, however, are not very well understood. All of this evidence combined shows that the OSM plays a pivotal role in the development and progression of prostate cancer and could be a valuable therapeutic target to improve stage outcomes in PC patients.

### 3.5 Testicular carcinoma

Testicular cancer is relatively rare, affecting only 1 in 250 males, however it develops in patients at a younger age, with an average diagnosis age at 33 years old (135). The role of OSM in testicular cancer has not been extensively studied, however *De Miguel et al.* in 1999 evaluated the presence of OSM in Leydig cells (cells in the testes responsible for testosterone production) as well as in various testicular carcinomas, including carcinoma-*in-situ* (CIS), germ cell tumors, and benign functioning Leydig cell tumors (148). OSM has been shown to cause a two-fold increase in the amount of Leydig cell progenitors, through stem Leydig cell differentiation in normal tissue samples. It was also found to be present in normal functioning and differentiated Leydig cells, therefore suggesting a role in normal Leydig cell differentiation and maintenance as shown by immunohistochemical staining (148, 149). OSM was also found within Leydig cells of patients with carcinoma-*in-situ* and in the parenchyma of neoplastic cells, however immunoreaction between cancerous and non-cancerous controls were similar, indicating OSM did not affect immune cell recruitment (148). Interestingly, functioning Leydig cell neoplasms showed a very strong immunoreaction to OSM, suggesting an upregulation of OSM in Leydig cell carcinoma may impact recruitment of immune cells (148). These preliminary studies evaluating OSM in testicular cancer suggest a possible role for OSM in Leydig cell differentiation and function of mature Leydig cells and recognize the presence of OSM in Leydig cell carcinoma and carcinoma in situ. However, no studies have yet to evaluate the tumorigenic properties of OSM in testicular cancer.

### 3.6 Colon

Colon cancer is the third most common type of malignancy and third leading cause of cancer-related deaths among men and women world-wide (135). The first connection of OSM in colorectal cancer was through discovering that the *OSMR* gene is highly methylated in non-invasive colorectal cancer patients, but not in normal controls, and has been suggested as a highly specific prognostic marker for colon cancer detection and severity of disease (150–152). In addition, a direct correlation between colorectal carcinoma (CRC) tumor grade and OSM expression level has been identified after examining the blood serum levels of OSM in colorectal cancer patients. High T staged CRCs (stages 3 and 4) have significantly higher levels (p < 0.001) of OSM present in the serum compared to low stage CRC as well as in healthy patient controls (153). Additionally, *Rajamaki et al.* identified hypomethylation and subsequent overexpression of *OSMR* in inflammatory bowel disease-associated CRC (IBD-CRC) patients, which may result in EMT of CRC cells and promote resistance to anti-TNFα therapies (mentioned in the Inflammatory Bowel Disease section) (154).

Camptothecin (CPT) is a chemotherapeutic agent frequently used in CRC and is aimed at the inhibition of topoisomerases (153). CPT has been shown to increase the expression of programmed death-ligand 1 (PD-L1) as well as other cytokines, including OSM (155). Examination of the TCGA colorectal cancer (COAD) and pan-cancer (PANCAN) database of ~4500 patients where high and low OSM expression was analyzed showing that high OSM expression was correlated with decreased patient survival (p < 0.001) further correlating the role of OSM in progression and metastasis in CRC (155).

### 3.7 Gastrointestinal

Every year, almost 1 million patients are diagnosed with gastric cancer worldwide, and almost 750,000 die, making it the second most common cause of cancer death worldwide (156–158). The role of OSM in gastric cancer has yet to be studied, but OSM has been shown to be overexpressed in pre-cancerous lesions and in gastric cancer (GC) when compared to normal gastric tissue, as well as in cancer-derived mesenchymal stem cells isolated from patients (159, 160). OSM expression in gastric high-grade intraepithelial neoplasia (HGIN) and early gastric cancer (EGC) tissues was significantly higher than that of low-grade intraepithelial neoplasia (LGIN) tissues based on expression profiling (p < 0.001) (159). RT-qPCR analysis of the OSM gene in EGC patients had a higher expression of OSM mRNA than that in HGIN (p < 0.05) and LGIN (p < 0.01), while immunohistochemical staining of OSM in LGIN was significantly lower than that in HGIN (p= 0.008) and EGC (p = 0.044) (159). These studies show that OSM could be a useful independent biomarker for possible staging of gastric cancer, and that the difference in OSM staining between HGIN and LGIN could be used as an early marker for gastric cancer.

OSMRβ has also been shown to be overexpressed in GC, highlighting the possibility of increased OSM-OSMR signaling in GC patients (161). Treatment with OSM increased proliferation and EMT *in vitro*. GC cells transfected with shRNA to knockdown OSMRβ expression had a reduction in the rate of proliferation (37.5%) as well as a reversal of EMT (161). These effects have been shown to be dependent on the activation of STAT3, FAK, and SRC through OSM OSM-OSMR signaling (161). Treatment with OSM increased GC tumor size and incidence of peritoneal dissemination *in vivo* with attenuation being reached through OSMRβ inhibition (161). These findings underline the effects of OSM within GC, resulting in increased proliferation, cell migration, invasion, and EMT dependent on OSM-OSMR signaling, as well as its potential as a T staging biomarker.

### 3.8 Pancreas

While pancreatic cancer has a low incidence rate due to lack of symptoms and early detection screening methods, pancreatic cancer has one of the worst prognosis rates, with 5-year survival at 12% for late-stage pancreatic cancer (135). OSM has been shown to play an important role in the progression of pancreatic ductal adenocarcinoma (PDAC), the most common form of pancreatic cancer originating from ductal cells within the pancreas by promoting EMT and by creating a more CSC phenotype in the pancreatic tumor microenvironment (162). Treatment of multiple human pancreatic cancer cell lines with recombinant human OSM (rhOSM) induced EMT via reduction of E-cadherin and induction of ZEB1 as well as upregulation of OSMRβ, leading to a positive feedback loop of increased OSM-mediated STAT3 signaling that maintains the malignant phenotype of these cells (162). *In vivo* analysis using xenografts of OSM producing PDAC cells showed that increased amounts of OSM in the TME caused greater primary tumor burden, increased metastatic spread, and led to a greater capacity to colonize the lungs (162). Co-culture models of human pancreatic cancer cells (HPAC) and human fibroblast overexpressing OSM also induced a CSC phenotype when compared to HPAC cells co-cultured with control fibroblasts (162). *Lee et al.* also found that OSM-OSMR signaling induces inflammatory fibroblasts within the TME in an *in vivo* PDAC model and promotes tumor growth and metastasis (163). A separate study also identified that similarly in breast cancer, OSM induces LOXL2 expression, subsequent collagen fiber alignment, and metastasis *in vivo* (164). Due to the low survival rate of PDAC patients, and lack of screening methods for early diagnosis, OSM, along with an array of other cytokines, have been shown to be overexpressed in the serum of pancreatic cancer patients and recent bioinformatics data has implicated OSM to promote radio resistance and poor prognosis in patients (163, 165, 166). This suggests OSM may be a useful clinical marker for diagnosis, and a therapeutic may increase survivability for pancreatic cancer patients. In contrast to these studies, *Nistal-Villan et al.* developed two oncolytic virus models encoding human OSM and, when administered to an aggressive orthotopic pancreatic cancer model in Syrian hamsters, was found to stimulate immune responses against cancer cells and had a significant anti-tumor effect (167). This work has not been examined further but may suggest a possible mechanism for recruitment of anti-tumorigenic immune cells to prevent cancer progression in pancreatic cancer.

### 3.9 Bladder

According to the American Cancer Society, in 2022 there are expected to be over 80,000 patients diagnosed with bladder cancer and nearly 17,000 deaths (135). While the role of OSM in bladder cancer has not been extensively studied, in 2019 *Deng et al.* published a study of 306 bladder cancer patients of Han residents within the Sichuan province of China and identified two novel single nucleotide polymorphisms (SNPs) within the promoter region of the *OSMR* gene (168). The two SNPs identified, rs2278329 and rs2292016 were identified in bladder cancer patients as well as healthy control patients. While rs2278329 allele variants showed no risk factors for bladder cancer progression, and patients with rs2292016 allele variants were associated with higher tumor grade and higher recurrence rate when compared to healthy control patients (168). Furthermore, a recent study performing whole exome sequencing of patients with bladder squamous cell carcinoma, also displayed significantly higher expression of OSM, OSMRβ, and IL-31, suggesting both OSM-OSMR and IL-31-OSMR signaling may impact bladder cancer progression (169). Recent evidence also suggests upregulation of OSM in metastatic bladder cancer patients (170). These studies illustrate that while the role of OSM and OSMRβ in bladder cancer has yet to be fully elucidated, OSMRβ allele variants may serve as a biomarker prognosis test for patients with bladder cancer, and therapeutics targeting OSMRβ may be a beneficial target for bladder cancer patients.

### 3.10 Lung

Lung cancer is the leading cause of cancer related deaths in both men and women, and the second most commonly diagnosed cancer (135). The current body of literature is conflicted on the role OSM plays in lung cancer progression. Some studies suggest OSM may repress lung cancer growth (171), but can promote lung cancer metastasis via activation of STAT3 and STAT5, thus increasing expression of tumorigenic factors such as tissue type plasminogen activator (tPA) (172, 173). Early work examining OSM in lung adenocarcinoma suggested it as a tumor promoter, including *in vitro* work showing OSM, and IL-6 to a lesser degree, as a potent inducer of human lung cancer differentiation. OSM combined TGF-β1 was also shown to regulate hyaluronan and may modulate lung cancer metastasis (174, 175). More recent work has also identified OSM as a tumor promoter *in vivo*. Adenovirus vector expressing mouse OSM induced a 13-fold increase in lung tumor burden and an increase in tumor size when compared to control cell lines. This effect was mitigated in OSMRβ KO mice, suggesting OSM-OSMRβ signaling is necessary (176). Other studies confirmed the pro-metastatic nature of OSM, demonstrating that it induces EMT in non-small cell lung cancer. Additionally, when lung cancer cells were co-cultured with cancer associated fibroblasts, there was an upregulation of phosphorylated-STAT3, OSMRβ, and LIFRβ, coupled with the downregulation of E-cadherin, suggesting an important role for fibroblasts in the activation of OSM signaling and the progression of lung tumors while protecting the cells from targeted therapies in an OSMRβ/JAK1/STAT3 dependent manner (177). This theory is also supported by *Wysoczynski et al.* who showed that lung cancer cells secrete an increased number of microvesicles when in the presence of stress factors like hypoxia and irradiation. Increased microvesicles lead to the activation of cancer associated fibroblasts and subsequent overexpression of pro-angiogenic factors such as OSM, IL-8, IL-11, VEGF, LIF, MMP-9, and tissue-type plasminogen activator (tPA) (172, 178). *Shien et al.* also analyzed patient data using the TCGA and PROSPECT lung adenocarcinoma databases and found a positive correlation of OSM, IL-6 and LIF in lung cancer patients, while also showing high OSMRβ expression had a significantly poorer prognosis compared to patients with a low OSMRβ expression (p = 0.0096 for recurrent-free survival), indicating that OSM and OSMRβ play a significant role in lung cancer (177). However, there is research that suggests OSM can suppress lung metastasis by inhibiting the EMT promoter SLUG, modulating mesenchymal-epithelial transition of lung cells by reducing EMT markers via STAT1 (179, 180). This combined research has not yet fully parsed out the mechanism of OSM in lung cancer, however patient data suggests a pro-tumorigenic and pro-metastatic role for OSM. Additionally, a study published by *Chen et al.* evaluated the expression of a short non-functioning form of OSMRβ, dubbed OSMRs, that is highly expressed in lung cancer patients, and acts a decoy receptor for OSM and thus resulting in mitigating OSMs oncogenic capabilities (181). This confounding factor may explain the contradictory results in lung cancer.

### 3.11 Brain

Brain cancer is a blanket term related to a variety of tumors based upon the cell type that becomes cancerous and includes both benign and malignant tumors. OSM has been shown to play a factor in a variety of brain tumor types including astrogliomas, astrocytomas, pituitary adenomas, glioblastomas, gliomas, medulloblastomas, and meningiomas (182–186, 189, 249). *In vitro*, OSM mediates tumorigenesis by activating STAT3 or STAT1 thus promoting expression of genes responsible for cell migration, ECM remodeling, and angiogenesis including, PLAU (plasminogen activator of urokinase), CHI3L3 (chitinase-like protein 1) and VEGF in several different human brain tumor cell types (183, 187, 249). In astroglioma cells, OSM induces an approximately three-fold increase in VEGF, while OSM and IL-1β together induce an approximately seven-fold increase of VEGF after 48 hours in astroglioma cells, in a STAT3 dependent manner (183). Additionally, OSM stimulation has been shown initiate the activation of the RelB/p50 proteins of the NF-κB pathway both *in vitro* and *in vivo*, perpetuating a tumor inflammatory environment in brain cancer cells (183). OSM-OSMR signaling mediated through STAT3, promoted MMP-9 upregulation over two-fold and increased the invasive potential of glioblastoma cells. OSM itself, however, did not influence tumor cell viability or proliferation (183). Two studies in fact suggest OSM may inhibit proliferation of glioma, astroglioma, and glioblastoma, although these studies have not been further evaluated (190, 191). Interestingly, *Jahani-Asl et al.* identified that OSMRβ is an essential co-receptor for EGFRvIII, and knockdown of OSMRβ strongly suppressed cell proliferation and tumor growth in mouse glioblastoma cells and human brain tumor stem cells in a xenograft mouse model (184). *Waters M. R., et al.* analyzed the correlation of OSM in brain cancer using brain tumor TCGA database and found OSM expression was most strongly correlated with poor glioblastoma multiforme (GBM; a heterogeneous mixture of cells containing brain tumor stem cells that are both tumorigenic and self-renewing) patient survival (182). They also discovered that OSM is produced in the brain solely by macrophages and microglia, and that chronic elevation of OSM leads to the progression of GBM (182). Macrophage-derived OSM has been shown to increase the mesenchymal like phenotype of OSM, mediated via STAT3 signaling both *in vitro* and *in vivo* (183, 186). Most recently, *Chen et al.* found that high OSM level is correlated with poor prognosis in several cancers, particularly with GBM, and found that OSM promotes migration and invasion of U251 glioblastoma cells while also exhibiting a more mesenchymal phenotype indicative of aggressive disease (192). OSMRβ has also been shown to be overexpressed in aggressive GBMs via STAT3 signaling and is correlated to a decrease in survival among patients (183, 184). Studies targeting OSMRβ and STAT3 suggest that a clinical therapeutic that disrupts OSM/OSMRβ/STAT3 signaling can repress brain tumor growth and increase chemoresistance in aggressive brain tumors (183, 249).

### 3.12 Squamous cell carcinoma

Cutaneous squamous cell carcinoma (cSCC) is the second most common keratinocyte malignancy, being responsible for 20% of skin-cancer deaths due to the lack of therapies (193). While OSM has not been extensively analyzed in all varieties of skin cancer, OSM has been shown to promote normal keratinocyte proliferation, migration, skin inflammation, and epidermal hyperplasia both *in vitro* and *in vivo* (52). OSM has also been shown to be overexpressed in cSCC patients, and overexpression of OSM *in vitro* and shown to induce STAT3 and ERK phosphorylation and activation, as well as increased proliferation and migratory capacity *in vitro* (194–196). Interestingly, during *in vivo* studies OSM was not found in the keratinocyte cells, but rather, it was found in large quantities at the periphery of the tumor due to infiltration of neutrophils, macrophages, and other inflammatory cells that secrete OSM in a paracrine fashion (194). OSM has also been shown to be highly expressed in keratoacanthoma; originally believed to be a benign form of skin cancer, but rare cases act in a similar form to skin SCC have been reported (195). In OSM-knockout mice, cSCC tumor volume was reduced by approximately 30% when compared to wild-type mice after one month, however there were still significant amounts of IL-6, IL-1β, IL-23α, CXCL1, IL-4, and IFNγ present in the tumor tissue compared to normal skin (194). The most understood environmental cause of cSCC is ultraviolet (UV) radiation (197). OSM signaling has been shown to suppress UV induce apoptosis of human keratinocytes and may be crucial towards early cancer progression *in vitro via* an increase in cell motility through ECM remodeling (197). This indicates that OSM may not only be crucial for cSCC progression, but also may lead to a higher incidence of squamous cell carcinoma, via repression of apoptosis in keratinocytes.

In addition to cSCC, OSM has also been implicated in oral squamous cell carcinoma (OSCC). In OSCC cells, treated with the known oral carcinogen arecoline, induced the expression of IL-6, STAT3, and c-Myc (198). The upregulation of c-Myc has been shown to suppress the expression of micro-RNA-22 (MiR-22) subsequently leading to an upregulation of OSM (198). This is reinforced by the observation that the expression of OSM and MiR-22 are inversely related (198). MiR-22 overexpression was able to suppress cell proliferation and migration by directly inhibiting OSM, suggesting the role of OSM in OSCC may be dependent on miRNA regulation (198).

Esophageal squamous cell carcinoma (ESCC) is the seventh most common malignancy in the world and is common among Asian populations (199). Due to difficulties of early screening, nearly half of patients are diagnosed as having locally advanced disease (200). The effect of OSM in ESCC has not been extensively studied, and preliminary reports suggest OSM plays a minor role. A recent study evaluating inflammation biomarkers in ESCC patients in Japan identified OSM as negatively correlated to the disease, while another study evaluating 173 cases in the ESCC TCGA dataset correlated OSM with a worse prognosis (201, 202). However, *Kausar et al.* identified a soluble form of OSMRβ (sOSMRβ) to be present in 9 out of 11 cell ESCC cell lines, and the presence of sOSMRβ protein was detected in the sera of patients. Furthermore, while high expression of OSM (94% of patients) was confirmed via IHC, full length OSMRβ was only detected in 23% of patients, suggesting that sOSMRβ may be acting as a neutralizing receptor for OSM in ESCC (203).

### 3.13 Kaposi’s sarcoma

OSM was first identified as a major growth factor in Kaposi’s sarcoma (KS) in 1992 when evaluating media from patients with AIDS-associated KS (4). Kaposi’s sarcoma is a relatively rare form of cancer but is endemic in several regions of the world and is estimated to be the leading cause of cancer incidence and mortality in several countries in Southern and Eastern Africa (199). When evaluating AIDS-KS cell lines, OSM was found to be a potent mitogen and autocrine growth factor (204, 205). Other studies showed OSM as a promoter of basic fibroblast growth factor (bFGF) which in turns promotes growth of Kaposi’s sarcoma and endothelial cells through activation of AP-1 response elements in the bFGF promoter (206, 207). It has also been proposed that KS-encoded cyclin K inhibits the anti-proliferative effects of OSM by directly inhibiting STAT3, although previous work suggests OSM signaling promotes growth via MAPK/ERK and JNK signaling (208, 209). Further studies also suggest that in KS, OSM and bFGF induce RAFTK, a focal adhesion kinase downstream of JNK, which acts as a convergence site for intracytoplasmic kinases and adapter molecules and increase cytokine signaling cascades and promoting cell growth (210). Additionally, AIDS-associated KS cells have been shown to express OSMRβ but not LIFRβ or IL-6 receptor, and inhibition of gp130 blocks the growth stimulating effects of OSM in AIDS-KS cells suggesting inhibition of OSM signaling may be beneficial strategy for patients with KS (211).

### 3.14 Miscellaneous sarcomas

Osteosarcoma (OSA) is the most common malignant bone disease in humans (135). *Fossey et al.* discovered that several OSA cell lines express OSM, OSMRβ, and gp130 receptor complex proteins, but interestingly not IL-6 or IL-6R. Activation of these receptor complexes occur with the binding of OSM leading to a time dependent increase in the levels of pSTAT3, pJAK2, and pSrc. While OSM does not increase proliferation of OSA cells (212–214), it does increase invasion via expression of glial fibrillary acidic protein (GFAP); a protein responsible for cytoskeletal reorganization in osteoblasts, MMP-2, cathepsin secretion and activity, as well as VEGF in a STAT3 dependent manner (212, 215–217). These features of OSM in OSA can increase the metastatic potential of OSA cells *in vivo*.

Chondrosarcomas (CSA) are difficult to treat, with chemotherapy and radiotherapy resistance, surgery remains the singular treatment option (218). Treatment with OSM induced cell cycle arrest in the S phase of murine SRC cell line *in vitro* and in G0/G1 of three other cells *in vitro* and is dependent on the JAK3/STAT1 pathway (218). Overexpression of OSM in the tumor cells, by adenovirus gene transfer, led to decreased tumor proliferation and enhanced apoptosis *in vivo* (218). These findings show that OSM treatment locally to the tumor environment of CSA could be a possible therapy to improve the prognosis of CSA patients.

Very little evidence exists for OSM and Ewing sarcoma (ES); however, the OSM gene has been shown to be differentially methylated in an ES microarray dataset (65% compared to healthy patients), although this did not significantly correlate with survival rate (219). Unlike OSA or CSA, OSM has been shown to increase the proliferation of ES cells in an OSMRβ/STAT3 dependent manner via upregulation of c-Myc (220). Based on the information given above, OSM inhibition would benefit CSA patients but burden OSA and ES patients.

### 3.15 Melanoma

Invasive melanoma accounts for only 1% of all skin cancer cases but is responsible for the vast majority of skin cancer deaths (135). Historically, OSM has been identified as a strong inhibitor of melanoma (221–223). Exogenous OSM has been shown to activate STAT3, STAT5b and the MAPK pathways via OSMRβ to strongly inhibit the proliferation of melanoma cells (221, 222, 224–226). OSM is also able to bind to collagens in a bioactive form and inhibit proliferation of A375 melanoma cells *in vitro* (227). Interestingly, OSM has also been shown to promote LIF expression, which could prolong the inflammatory effects of OSM (228). OSM also increases expression of membrane bound ICAM-1 *in vitro*, which also may suggest higher immune surveillance in human myeloma (229).

As melanoma reaches an advanced stage, cells appear to become resistant to the inhibitory effects of OSM (225). This has been shown *in vitro* and *in vivo* to be partly caused by the constitutive expression of suppressor of cytokine signaling-3 (SOCS-3) mRNA and subsequent high level of SOCS-3 protein (230). Accompanying the increase in SOCS-3 mRNA/protein is a downregulation of the OSMRβ subunit, due to a decrease in the amount of histone acetylation in the promoter region of the OSMRβ gene (231). Paracrine signaling of OSM in antigen-negative melanoma cells to antigen-positive will lead to antigen-silencing, possibly affecting the outcome of antitumor vaccine immunotherapies (232). OSM sensitivity in human melanoma cells also has importance for tumor-infiltrating lymphocyte (TIL) treatment of stage 3 melanoma (233). Patients that were unresponsive to OSM expressing TILs due to phosphorylation defects of STAT3 on Ser-727, as well as activation of AKT on Ser-473, were shown to have an increased resistance to OSM anti-proliferative activity (233). The development of OSM resistance in melanoma cells has a significant role in creating a more aggressive phenotype, which appears to be specific to melanoma. This could be treated by increasing the amount of OSM ligand in the system through the use of an OSM therapeutic.

## 4 Therapeutic intervention of OSM

As mentioned in the previous sections, developing an effective targeted therapeutic against OSM signaling could be crucial for the treatment of numerous diseases, including a variety of cancers. While several therapies have been developed and approved that target IL-6 and other IL-6 family members, currently no FDA approved treatments exist for OSM. This section will outline previous and current strategies for developing effective therapeutics against OSM and OSM receptor, including unique strategies that are in early stages of testing. The specific drugs and patents for drugs against OSM and OSMRβ are described in **Table 3**.

**Table 3:** Anti-OSM and Anti- OSMRβ Therapeutics.

Drug Name/Patent Number	Company /University	Type	Disease	Progress	References	
Anti-OSM therapeutics					
GSK315234	GlaxoSmithKline	mAb	Rheumatoid Arthritis	Stage II clinical trial (*failed*)	(236)
GSK233081	GlaxoSmithKline	mAb	Systemic Scleroderma	Stage II clinical trial (*failed*)	(237–240, 250)
US7858753B2	GlaxoSmithKline	mAb	Non-specific	Pre-clinical	(240)
US6706266B1 WO2020127884A1	GlaxoSmithKline Universite de Poiters	Aptamer mAb	Rheumatoid Arthritis Inflammatory Skin Disease/Cancer	Pre-clinical Pre-clinical	(240) (251)
US20170327573A1	University of Padua	Broad Therapeutic	Diabetes/Cardiovascular diseases	Pre-clinical	(252)
US9550828B2	Boise State University	SMI	Cancer	Pre-clinical	(253)
Anti-OSMR therapeutics					
US9663571B2	Kiniksa Pharmaceutical	mAb	Atopic Dermatitis	Pre-clinical	(254)
US10493149B2	Kiniksa Pharmaceutical	mAb	Non-specific	Pre-clinical	(255)
WO2013168829A1	Wakayama Medical University	mAb	Atopic Dermatits/Puritis	Pre-clinical	(256)
US20090300776A1	Universitie D’angers	siRNA	Inflammatory Skin Diseases	Pre-clinical	(257)
US7572896B2	Raven Biotechnologies	mAb	Cancer	Pre-clinical	(258)
WO 2010139742A1	Max Plank Society	Broad Therapeutics	Heart Failure	Pre-clinical	(259)

### 4.1 Anti-OSM therapeutics

**Figure 2:**
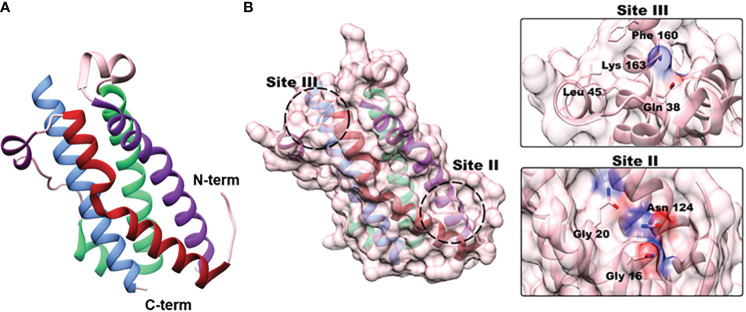
OSM structure and receptor binding sites. **(A)** OSM is a four α-helical bundle protein consisting of helices A (red; aa 10 –37), B (green; aa 66-90), C (purple; aa 106-131), and D (blue; aa 159-185) (truncated X-ray crystallography PDB:1EVS). **(B)** Site-directed mutagenesis experiments have identified two separate binding sites responsible for receptor-ligand interactions. Site II is located near the N-terminal region in helices A and C, and three amino acids (Gly-16, Gly-20, Asn-124) have been identified as crucial for OSM interaction with gp130. Site III is located in the A- B loop with a small portion of the D helix (dark purple) that is responsible for binding of OSM to LIFRβ and OSMRβ. Substitution experiments with OSM and LIF revealed that Lys-163, and Phe-160 are required to bind to both receptor complexes, but Tyr-34, Gln-38, Gly-39 and Leu-45 are specifically needed for interactions with OSMRβ.

In 2000, *Deller et al.* published the first molecular structure for OSM, paving the way for the development of potential anti-OSM therapeutics (5). OSM’s tertiary structure consists of four α-helical bundles (helices A-D; **Figure 2A**) and has two distinct sites responsible for receptor complex binding (**Figure 2B**) (5). The Site II motif, consisting of regions of helices A and C, is responsible for OSM’s binding to gp130. Site-directed mutagenesis has revealed four amino acid residues: Gln-16, Gln-20, Gly-120, and Asn-124, shown to be the primary residues responsible for this interaction (5, 16). The Site III epitope, located within a loop between helices A-B (**Figure 2A**), and near the N-terminal end of helix D, is the primary site by which OSM binds to OSMRβ and LIFRβ. This region is highly conserved for both OSM and LIF, thus making it difficult to generate a specific therapeutic against OSM. However, recent research efforts have shown that OSM possesses a unique amino acid composition that is necessary for specific interactions with OSMRβ. Alanine-scanning experiments and substitution experiments comparing OSM and LIF revealed that Tyr-34, Gln-38, Gly-39, and Leu-45 (in AB loop) and Pro-153 (in helix D) are responsible for OSMRβ binding, while Phe-160 and Lys-163 of D-helix are necessary for interaction with both OSMRβ and LIFRβ (16, 234, 235).

The pharmaceutical giant GlaxoSmithKline (GSK) has supported the production of two separate anti-OSM neutralizing antibodies, both of which target the Site II region of OSM, and to date, these are the only anti-OSM therapeutics to advance into clinical trials. GSK315234 is a humanized anti-OSM IgG monoclonal antibody developed for the treatment of patients with active rheumatoid arthritis (RA), designed to bind to the Site II region of OSM and prevent dimerization with gp130 (236). A phase two clinical trial was initiated with the goal of investigating the safety, pharmacokinetics (PK), and pharmacodynamics (PD) of GSK315234 in patients with RA. The study contained a total of 135 patients with RA, was divided into four groups (double-blind, placebo-controlled, and randomized, and evaluated the following: i) an intravenous (IV) method of delivery, ii) a subcutaneous (SC) method of delivery, iii) a single dose delivery (single versus multiple delivery) and iv) a multiple dose delivery, all over a period of 154 days. Patients selected to participate in the study were required to have active RA with a Disease Activity Score 28 (DAS28) of > 4.2 at screening. DAS28 is a composite score analyzing the number of swollen/tender joints (that includes 28 joints), as well as examining concentration of erythrocyte sedimentation rate (ESR) and C-reactive protein (CRP) in the blood stream. Patients selected to participate in the study were also required to have had previously received at least three months of treatment with methotrexate.

Overall, evidence from this study suggests that repeated dosing with GSK315234 did not demonstrate statistically significant efficacy. While there appears to be minimal toxicity in patients who received GSK315234, the monoclonal antibody exhibited poor binding affinity (2.5 nM) and a rapid off-rate (1.73 x 10^3^) when compared to the higher affinity of OSMRβ (approximately 150 pM). Interestingly, patients in the group which received a single 3 mg/kg dose of GSK315234 by IV displayed a statistically significant reduction in DAS28 score compared to the placebo group (p-value <0.05 at days 56, 84, and 91), as did the patients that received a 10 mg/kg dose by IV (at day 84). However, groups receiving larger single doses (20 and 30 mg/kg IV), repeated dosing (6 mg/kg IV), or SC injection (500 mg) exhibited no significant difference in clinical score. Due to the high off-rate and binding affinity, as well as the poor significance and inconclusive results in the study, GSK315234 clinical trials were halted.

Another GSK anti-OSM monoclonal antibody, GSK2330811, has entered clinical trials for treatment of systemic scleroderma (237, 238). A phase one, randomized, double-blind, placebo-controlled SC administered clinical trial with 30 healthy subjects showed a favorable safety profile in participants. Patients were divided into 6 groups, with patients given either placebo or varying concentration of a single SC dose of GSK2330811 (0.1, 0.3, 1, 3 or 6 mg/kg respectively). No clinically relevant change from baseline laboratory values were observed in any of the groups, and GSK2330811 exhibited pharmacokinetics over all five of the dose ranges with a binding affinity estimated at approximately 0.58 nM (95% CI 0.455, 0.710). This drug has recently finished evaluation in phase two clinical trials for patients with diffuse cutaneous systemic scleroderma involving 35 patients randomized to placebo receiving 100 mg or 300 mg treatment subcutaneously every other week for 12 weeks. There were no clinical differences between GSK2330811 and placebo groups. Additionally, all patients in the 300 mg treatment group reported at least one adverse effect; including decreased hemoglobin, anemia of varying severity, decreased platelet counts, decreased neutrophil counts, and thrombocytopenia (239). A separate clinical trial (NCT04151225) was also initiated with GSK2330811 for patients with Crohn’s disease; however, the study was halted by an internal sponsor before patient enrollment began due to a potentially narrow therapeutic window (238, 240, 250).

In addition to the previously mentioned monoclonal antibodies that have advanced to clinical trials, GlaxoSmithKline possesses a patent (US7858753B2) (240) for another anti-OSM antibody. While the previous ones were designed to specifically interact with Site II of OSM (**Figure 2**), this is designed to inhibit OSM/gp130 interaction without directly binding to any amino acids within Site II. Instead, it is designed to interact with amino acids; Pro82, Ser83, Glu84, Leu90, Gly94, Pro112, Gln115, Asp122, Leu123, and Cys152 of OSM. It is unclear how this antibody interferes with OSM/gp130 dimerization, but further studies may reveal its mechanism. Within a separate patent (US6706266B1) (240), GlaxoSmithKline also has RNA aptamer designs that are highly specific towards OSM with a K_D_ at approximately 7 nM; however, both of these patents have yet to be tested *in vitro, in vivo*, or through clinical trials.

Researchers at the Universite de Poiters have also developed a patent (WO2020127884A1) relating to specific binding proteins, such as an antibody, that binds specifically to OSM to inhibit interaction with OSM and gp130, and/or LIFR, to be used with any disease associated with elevated levels of OSM, with a particular interest in inflammatory skin diseases and cancer (251).

A different patent, submitted by the University of Padua (US20170327573A1) (252), is designed to develop an anti-OSM therapeutic that will increase mobilization of bone marrow stem cells in patients with diabetes. This patent allows for the design of a variety of inhibitors that would inhibit OSM production or OSMRβ signaling at the cellular level such as enzyme inducers, an enzyme or receptor inhibitor, a ligand for a receptor, a compound that is toxic for cells, or an antisense RNA.

Utilizing a separate approach, Boise State University has submitted a patent (US9550828B2) (253) developing small molecule inhibitors against the Site III region of OSM, preventing OSM binding to OSMRβ. The compounds proposed in this patent are designed to reduce tumor cell detachment, invasion, and metastasis. Recently, a specific small molecule inhibitor named SMI-10B was characterized and shown to bind to specific amino acids within Site III of OSM via HSQC-NMR (260), and was subsequently confirmed by an independent group using molecular dynamics simulation (261). To date, this is the only patent designed for small molecule inhibition of OSM.

### 4.2 Anti-OSMRβ therapeutics

As outlined in **Figure 1**, OSM interacts with gp130, which then results in the dimerization of both LIFRβ and OSMRβ, however it is thought that human OSM binds with a much stronger affinity to OSMRβ than to LIFRβ. Another cytokine, IL-31, also uses OSMRβ as part of its receptor complex (along with its receptor IL-31RA), making OSMRβ, specifically, a beneficial target to inhibit OSM as well as IL-31 signaling (262, 263). Challenges exist for this strategy due to the unknown structure of OSMRβ. Recent work utilizing computational *in silico* analysis and homology modeling of the structurally similar LIFRβ has provided framework for structural modeling of the OSM-OSMR complex, providing more detailed information for those designing therapeutics against OSMRβ (257). Furthermore, with the recent advancement in molecular modelling, particularly with *AlphaFold*, a predicted structure for OSMRβ has been created, which may help pave the way for specific targets against the receptor protein (264, 265). Currently, no therapeutics targeted against OSMRβ are clinically available, and to date, none have advanced into clinical trial stages. However, several patents have been submitted for a variety of compounds designed as inhibitors of OSMRβ and will be outlined below (see **Table 3**).

As highlighted previously, OSM has been shown to play a role in various inflammatory skin diseases. To combat this, Kiniksa Pharmaceutical currently has two patents, all describing monoclonal antibodies, that are designed to inhibit OSMRβ in inflammatory skin conditions. The first (US9663571B2) (254) is designed for the treatment of atopic dermatitis and chronic puritis in patients who have yet to receive treatment with a corticosteroid, or for patients with serum IgE levels lower than 300 IU/mL. Both OSM and IL-31 have been linked to atopic dermatitis. OSMRβ, which is part of the receptor complex for both proteins, makes it a desirable target to inhibit both OSM and IL-31 signaling. This patent outlines three separate antibodies that describe an IC_50_ range between 157 pM and 1.35 nM and an average K_D_ of 0.2 nM. Kiniksa Pharmaceutical’s second patent, (US10493149B2) (255) is also a monoclonal antibody is for unspecified diseases. Furthermore, two other patents have been developed for inflammatory skin disorders. Wakayama Medical University has submitted a patent (WO2013168829A1) (256) for a monoclonal antibody against OSMRβ designed to inhibit both OSM and IL-31 induced inflammation in patients with atopic dermatitis. Universitie D’angers has also written a patent (US20090300776A1) (257) for a small interfering RNA (siRNA) that is designed to inhibit OSMRβ mRNA expression in keratinocytes that would subsequently result in repressed inflammation in a variety of inflammatory skin diseases. This patent also encompasses molecules designed to inhibit a variety of cytokines linked with keratinocyte-mediated inflammation, including OSM, IL-17, TNFα, IL-31, and IFN-y.

Additionally, two pharmaceutical companies have patents targeting OSMRβ in both cancer and heart disease. Raven Biotechnologies has developed an anti-OSMRβ antibody (US7572896B2) (258) designed for diagnosis of human cancers with high OSMRβ expression, as well as treatment for a variety of human cancers. Mouse model experiments using human ovarian and lung cancer cells indicate this antibody is effective at reducing proliferation both *in vitro* and *in vivo.* Furthermore, the Max Plank Society has written a patent (WO 2010139742A1) to develop an anti-OSMRβ therapeutic for the treatment and/or prevention of heart failure (259). The patent is broad in nature, and is written to encompass an aptamer, siRNA, shRNA miRNA, and/or ribozyme. While current efforts for an anti-OSM or anti-OSMRβ drug have not yet succeeded in making it through clinical trials, it is clear that a therapeutic is needed for a variety of diseases. Developing novel therapies that target OSM or OSMRβ with high specificity and low toxicity will hopefully provide the necessary therapeutics for patients with abnormal OSM or OSMRβ expression.

## 5 Discussion

Throughout the course of this review, we have shown the important role OSM plays in a variety of diseases including many types of cancer. OSM activates several signaling pathways, frequently leading to inflammation, migration, or regeneration and differentiation (19–23). OSM can be produced and secreted by many different cell types, mostly activated immune cells such as macrophages and neutrophils, to intensify some inflammatory diseases, as shown in **Table 1** (1, 2, 24). Several studies have linked OSM overexpression with an overall worse prognosis for a variety of diseases, including arthritis, IBD, and most recently COVID-19 (25, 26, 39, 42, 90). However, OSM expression has the possibility to be beneficial to healing injuries, particularly regarding the CNS, bone, liver, heart, and general external skin wounds (33, 37, 64, 68–72, 75–77, 83–85, 92). Negative effects due to chronic expression of OSM may outweigh its positives, yet inhibiting the signaling systemically may cause issues within other physiological processes.

The role of OSM in cancer has also been mysterious (see **Table 2**). While OSM was initially discovered as an inhibitor of cancer cell growth in melanoma cells (221–223), OSM has been shown to play an important role in cancer progression. In fact, while OSM expression has been shown to repress tumor growth in some cancer types it also may promote tumor growth in other types (15, 102, 103, 161, 162, 171). The specific mechanisms by which OSM operates under different cancer subtypes has yet to be fully explored. However, increased OSM signaling has been shown to increase the proliferation, motility, and metastatic potential of multiple cancers (36, 109, 140, 143, 162, 177, 183, 186, 248). It is also interesting to consider OSM or OSMRβ as a possible biomarker for certain types of cancers such as cervical, colon, GI, and pancreatic cancer (153, 159, 165, 242). Overexpression of OSM and/or OSMRβ is seen more commonly in a multitude of advanced tumors and is linked to decreased patient survival in several cancer types, including breast, cervical, colon, pancreatic, lung, and brain cancer (109, 153, 165, 177, 192, 242). Patients in a clinical setting could benefit greatly from an anti-OSM therapeutic, but the market remains empty for oncologists and their patients.

There are several therapeutics currently in development designed to inhibit the OSM signaling cascade, some by binding to OSM and others by binding its receptor, OSMRβ (see **Table 3**). The current strategies being implemented represent diverse and novel approaches to develop the most effective inhibitor. Significant inhibition of cytokines has proven to be a challenge clinically. To date, IL-6 remains the only member of its family to have FDA approved clinical therapeutics (266, 267). Targeting OSM, however, has proven to be more challenging. While two monoclonal antibodies against OSM have been the only potential therapies to reach clinical trial stages, both struggled with poor binding affinity and lack of clinical significance (236, 237). It is possible an alternative strategy is needed for an effective anti-OSM therapeutic. Monoclonal antibodies tend to have lengthy half-lives (on the order of days or weeks) that may affect normal inflammatory response mechanisms in cases of infection or injury, and serious wound healing might require pausing therapy (268). In the case of GSK2330811, the half-life was reported to be approximately 24 days (237). Furthermore, both clinical trials of GSK315234 and GSK233081 reported long-term accumulation of OSM-mAb complexes, directly resulting from their long half-life, which in combination with rapid off rate and poor binding affinity, may result in lengthening active OSM in the bloodstream of subjects (236, 237). The wide variety of techniques being implemented, including small molecule inhibitors, aptamers, and other biologics, may eliminate long-term issues with accumulation, provide a highly specific and minimally toxic therapeutic for patients, and allow for therapy to be paused when necessary.

In addition to targeting OSM, several drugs are currently in development to inhibit OSMRβ rather than OSM. While targeting OSMRβ is a valid strategy, and several attempts have been initiated, none of them have entered into clinical trials. As a target, OSMRβ possesses its own unique challenges, lacking a completed crystalized structure; forcing medical chemists to rely on computational modeling for targeting amino acids necessary for OSM/OSMRβ (269). Furthermore, targeting the other OSMR subunit gp130 itself is a risky venture due to its diverse role in all IL-6 family cytokine receptor complexes, although one group is investigating inhibition of gp130 for specific cytokines in the context of inflammatory diseases and multiple cancers (270–272).

## 6 Conclusion

Throughout this review, we have outlined the evidence for identifying OSM as a therapeutic target for numerous diseases, as well as a variety of cancers. While efforts have been initiated to develop clinical therapeutic for patients, to date, none exist. Creating an anti-OSM or an anti-OSMRβ therapeutic is a much-needed venture for patients and clinicians alike, and work must be continued to synthesize and generate an FDA approved therapeutic.

## Author contributions

CW contributed to conceptualization, original draft reparation, creation of figures and tables, and final review & editing. CP contributed to conceptualization and original draft preparation, and creation of figures and tables, and final review & editing. DL contributed to conceptualization and original draft preparation, and creation of figures and tables. CJ contributed to conceptualization, original draft reparation, creation of figures and tables, and review & editing, supervision of the project, and funding acquisition. All authors contributed to the article and approved the submitted version.
